# Demand-Sensitive Time Allocation in Digital Reading: Evidence from 25 Education Systems

**DOI:** 10.3390/jintelligence14100264

**Published:** 2026-10-16

**Authors:** Yan Li, Jinkai Zhang, Gina S. Rhee

**Affiliations:** 1 School of Culture and Communication, Bozhou University, Bozhou 236800, China; bzszliyan@163.com; 2 School of Literature and Communication, Minzu Normal University of Xingyi, Xingyi 562400, China; kelvinsoul@naver.com; 3 The Catholic College, The Catholic University of Korea, Bucheon 14662, Gyeonggi-do, Republic of Korea

**Keywords:** digitalPIRLS, demand-sensitive time allocation, individual differences, cross-item generalization, reading comprehension

## Abstract

Digital assessments record item-visit durations and revisiting. These traces can reveal responses to task difficulty, but achievement associations do not establish stable person-level measures. We linked digitalPIRLS 2021 process, response, and achievement data across 25 education systems, obtaining 121,908 students with usable process records and 116,206 complete cases for the primary achievement models. Item difficulty was estimated from other students in five groups, excluding each target student’s own responses. We measured how each student’s first-visit time and revisiting changed with difficulty. More difficult items elicited longer first visits in all 25 systems, whereas the average difficulty effect on revisiting was weak. A one-standard-deviation increase in time adaptation was associated with 19.92 overall-reading points, compared with 3.19 points for revisit adaptation. Effect magnitudes varied substantially across systems. Repeated analyses of separate item halves showed limited stability for time adaptation (0.317) and moderate stability for revisit adaptation (0.529), after correction for test length. Time adaptation estimated from four fifths of the items remained associated with performance on the remaining fifth in all 25 systems. These findings support time adaptation as a task-responsive behavioral indicator within digitalPIRLS. They do not establish a highly reliable trait score or identify a single cognitive mechanism.

## 1. Introduction

Digital assessments record more than whether an answer is correct. They also record how long students remain on an item, whether they leave and return, and how they navigate through the test. These traces make it possible to ask a simple but important question: do students change what they do when a task becomes harder? A second question is equally important for individual-differences research: does a process measure that is related to achievement also rank students consistently when a different set of items is used? The two questions are not the same. A measure can be informative about behavior in a task while still being too item-dependent to serve as a stable person-level score (Cronbach et al., 1972; Brennan, 2001; Goldhammer et al., 2021).

Response time illustrates this distinction. Spending more time can reflect deeper processing, difficulty, caution, strategy, or inefficient dwelling; spending less time can reflect skill or rapid guessing. Its meaning therefore depends on the item and the testing situation rather than time alone (Kyllonen & Zu, 2016). Recent work similarly shows that systematic changes in response time and within-person variability can contain cognitive information instead of being treated only as measurement noise (Junghaenel et al., 2023; Vaughan & Birney, 2023).

The present study therefore examines observable behavior rather than assuming that time traces directly reveal a specific cognitive mechanism. We ask whether students increase first-visit time or return to items more often as empirically estimated item difficulty increases, whether those person-specific patterns are associated with reading achievement, and how stable they are across different item samples. We refer to the first pattern as time adaptation and the second as revisit adaptation. These measures are consistent with demand-sensitive resource allocation, but first-visit time alone cannot distinguish attentional investment from decoding difficulty, strategy use, interface effects, or other processes.

Digital reading provides a particularly informative context for this question. Reading comprehension requires the coordinated operation of lexical access, information retrieval, inference, discourse integration, and monitoring (Kintsch, 1988; Perfetti & Stafura, 2014). Items differ substantially in the cognitive demands they impose, but the observation that difficult items often take longer is not, by itself, theoretically informative. The more consequential issue is that average response time collapses behavior across heterogeneous task conditions into a single-level parameter and therefore cannot distinguish general slowness from conditional responsiveness to demand. We therefore ask whether a student’s process behavior changes systematically with independently estimated item difficulty and whether that task-responsive relation simultaneously shows criterion association, person-level stability, and cross-item generalization.

Research on digital reading already provides substantial process evidence. Small-sample tracking studies show that children with similar comprehension outcomes may follow markedly different screen-reading paths and that rereading, scrolling, and tool use do not form a single optimal pattern (Shimizu et al., 2024). Digital features such as scrolling and hyperlinks can also alter cognitive load and reading behavior (Krenca et al., 2024). Large-scale assessment research has used response time to identify rapid responding, engagement profiles, and aberrant behavior and has leveraged highlighting and navigation traces to explain between-system differences (Anghel et al., 2024; Leng & von Davier, 2025; Anghel & von Davier, 2025). Together, these studies demonstrate the explanatory potential of process data while exposing an unresolved problem: the same indicator may show stable group-level associations with achievement while remaining insufficiently stable as a person-level score.

Psychometric research on intelligence and cognitive assessment is increasingly addressing the interpretation of process data directly. Recent studies have modeled nonlinear relationships between response accuracy and response time and have incorporated effort-related process behavior into validity analyses (Chen et al., 2026; Guo et al., 2026). The contribution of the present study therefore does not lie in using response time per se. It lies in integrating task-conditioned process slopes, cross-system criterion associations, person-level reliability, and generalization to nonoverlapping items within a single evidential framework.

We use demand-sensitive cognitive resource allocation as a theoretical interpretation of the observed process patterns, not as a directly observed mechanism. Operationally, demand-sensitive first-visit time allocation (time adaptation) captures how strongly a student increases first-visit time as empirically estimated item difficulty rises, whereas demand-sensitive revisiting (revisit adaptation) captures whether returns are increasingly concentrated on more difficult items. Both indicators are task-conditional and distinct from average speed and the overall revisit rate. Because performance-based item difficulty can also reflect linguistic, curricular, item-format, and instructional characteristics, we treat it as a cross-fitted empirical proxy for task demand rather than a pure measure of intrinsic cognitive demand. The study evaluates four separable forms of evidence: task responsiveness, association with reading achievement, stability of person rankings across difficulty-balanced item subsets, and criterion association across mutually nonoverlapping item sets.

The PIRLS 2021 International Database, Edition 2, provides unusual leverage for this design. The public digitalPIRLS files include item-level first-visit time, total time, and number of visits, together with item responses, complex-sample weights, Jackknife replicate weights, and multiple plausible values. The study covers 25 education systems with usable process data and 121,908 students with usable process records, including 116,206 complete cases in the primary achievement models. Item difficulty is estimated with five-fold cross-fitting so that each target student’s own responses do not contribute to the difficulty values used to construct that student’s process slopes. System-specific estimates are then synthesized by random-effects meta-analysis, and leave-one-education-system-out analyses evaluate directional transportability. Model diagnostics, 100 repeated split-halves, booklet sensitivity analyses, and five-fold item-set holdout analyses further separate task responsiveness, criterion association, person-level stability, and cross-item generalization.

Relative to prior work, the theoretical increment is concentrated in three connected steps. First, item difficulty is used as a cross-fitted empirical demand gradient to estimate within-person conditional resource allocation. Second, the cross-system reproducibility of process effects is evaluated separately from the cross-item dependability of person scores. Third, nonoverlapping item holdout analyses test whether process indicators carry criterion information beyond the items from which they were constructed. This design responds directly to calls in individual-differences research to evaluate cognitive processes, score reliability, and interpretive boundaries together rather than treating them as interchangeable forms of evidence (Vaughan & Birney, 2023; Brysbaert, 2024).

### 1.1. From Average Speed to Task-Responsive Process Indicators

Response time is highly available and highly ambiguous. Longer times can reflect deeper processing, hesitation, comprehension difficulty, low automaticity, or unproductive dwelling; shorter times can indicate skill, but they can also reflect rapid guessing. Reviews of large-scale assessment process data show that item difficulty, item position, cognitive complexity, and interface features all alter time distributions and that ability–time relations depend on task structure (Anghel et al., 2024). Cho et al. (2024) likewise showed that time indicators must be interpreted in relation to item function when comparing paper-based and digital reading. Average time is therefore useful as a general behavioral level but is less suitable as a direct indicator of adaptive resource allocation. This is consistent with the speed–level distinction in intelligence measurement: response time is not a unitary index of ability speed, because its meaning depends jointly on accuracy, item demands, and individual strategy (Kyllonen & Zu, 2016; Guo et al., 2026).

Conditional process indicators offer a different route. Goldhammer et al. (2021) argued that valid interpretation of log-data indicators requires an explicit evidential link among the target construct, task properties, and behavioral mechanism. In the present study, reading-item difficulty serves as an empirical demand proxy rather than a direct measure of cognitive load: it can reflect cognitive complexity but also linguistic, curricular, and item-specific characteristics. If students respond to perceived task requirements by changing their behavior, first-visit time or revisiting should covary systematically with that cross-fitted difficulty gradient. The within-person difficulty–first-visit-time slope therefore has a clear descriptive meaning: a steeper slope indicates that the student adds more initial processing time as empirically estimated difficulty rises. The difficulty–revisit slope analogously captures whether re-engagement is increasingly concentrated on more difficult items. These indicators reduce the influence of average speed and general operating habits, while their cognitive interpretation remains a hypothesis to be evaluated rather than an observed mechanism.

The closest precedent is Junghaenel et al. (2023), who separated mean response time, systematic response-time adjustment, and residual within-person variability in a large web-based survey. Systematic adjustment relative to item time intensity carried more information about cognitive ability than mean response time alone. That result supports the general proposition that conditional time adjustment can be cognitively informative. The present study extends this logic to reading assessment by using cross-fitted item difficulty as the demand gradient, examining both first-visit time and revisiting, and evaluating transportability across 25 education systems, split-half stability, and item-set holdout generalization.

### 1.2. RESOLV, Task Models, and Cognitive Resource Allocation

The RESOLV framework conceptualizes task-oriented reading as an interaction among context representation, task representation, and action selection. Readers form task models based on reading goals, success criteria, and perceived task requirements, and these task models guide information search, dwell time, checking, and answer production (Rouet et al., 2017). When an item requires more complex inference or evidence integration, the task model can prompt additional processing, renewed text search, or answer checking. Time and revisiting in digital environments therefore provide observable behavioral windows onto the consequences of task representation.

Classic work on study-time allocation likewise emphasizes selective resource allocation. Learners adjust study time across materials according to monitoring outcomes, goals, expected utility, and time constraints (Nelson & Leonesio, 1988; Ariel et al., 2009; Dunlosky & Ariel, 2011). The regulatory principle does not imply that stronger students spend more time on every item. The key question is whether additional time is selectively concentrated on higher-demand tasks. Recent digital-reading studies have increasingly examined reviewing time, adaptive navigation, and trace-based self-regulation (Ronqui Leites et al., 2024; Naumann et al., 2025; Mañá et al., 2026). We therefore treat time adaptation as a demand-responsive indicator of initial resource allocation and revisit adaptation as an indicator of subsequent re-engagement.

More general theories of cognitive control and effort allocation also caution against treating resource investment as a mechanical function of task difficulty. Cognitive effort carries subjective and opportunity costs, and individuals must decide whether and how much control to allocate as a function of demand, expected benefit, and effort cost (Shenhav et al., 2017; Kool & Botvinick, 2018). Accordingly, the theoretical significance of time adaptation is not that difficult items take longer, it is that it quantifies whether a given student selectively changes processing intensity along a cross-fitted empirical difficulty gradient. This pattern is consistent with dynamic cognitive-control accounts, but the time trace itself cannot identify which underlying process produced the adjustment.

### 1.3. Initial Allocation and Revisiting as Distinct Regulatory Stages

First-visit time and revisiting occur at different behavioral stages. First-visit time captures the duration of the initial item visit and may be consistent with resource investment during initial processing, but it does not directly measure that investment. Revisiting occurs after the student has left the item and may involve subsequent monitoring, perceived uncertainty, evidence search, or answer revision. Because the cognitive prerequisites differ, the two behaviors should not be collapsed into a single engagement measure. Shimizu et al. (2024) showed that children can achieve comprehension through different rereading and navigation paths and that frequent returns are not uniformly efficient. Anghel and von Davier (2025) similarly showed that the quantity and quality of highlighting behavior need to be distinguished. Recent work with fifth-grade readers also indicates that metacognitive monitoring is distinguishable from vocabulary, fluency, and cognitive flexibility, rather than being a simple byproduct of comprehension performance (Markovich et al., 2026).

The present analyses therefore control both mean first-visit time and overall revisit rate. Time adaptation asks whether difficult items receive disproportionately more initial processing time; revisit adaptation asks whether additional returns are disproportionately concentrated on difficult items. This decomposition isolates conditional allocation beyond average behavioral levels. Because revisiting requires additional monitoring and decision making, its average difficulty effect may be more context-dependent, whereas first-visit time may respond more consistently to item demand.

### 1.4. Does Strong Criterion Association Imply a Stable Individual Trait?

Process-indicator research must distinguish two questions that are often conflated. The first is whether an indicator is associated with a theoretically relevant external criterion. The second is whether the indicator can preserve relative standing across task samples when used as an individual score. The first concerns criterion-related evidence; the second concerns score dependability. Generalizability theory emphasizes that observed scores always depend on a universe of admissible observations, and that interpretation must narrow when item-sampling variance is large (Cronbach et al., 1972; Brennan, 2001). Kane’s (2013) argument-based approach to validity similarly stresses that score interpretation and use must remain commensurate with the evidence supporting them.

Reliability becomes particularly salient when experimental or process tasks are used for individual-differences inference. Hedge et al. (2018) described a reliability paradox in which an experimental effect can be highly robust at the group level but poorly suited to ranking individuals because true between-person variance is limited. Large-scale work on self-regulation measures has likewise found that behavioral tasks often show lower test–retest reliability than questionnaire scores (Enkavi et al., 2019). Behavioral process measures should therefore report reliability directly rather than inferring trait-like stability from significant group effects (Parsons et al., 2019).

This issue is especially important for process slopes. Time-adaptation slopes are estimated from a finite number of items. Empirical-Bayes shrinkage can reduce sampling noise, but different item combinations may still alter person rankings. An indicator can therefore show a stable average process effect across education systems and a strong association with reading achievement while exhibiting only limited person-level cross-item stability. In that case, the more defensible interpretation is a task-responsive process marker rather than a highly reliable trait. Conversely, high split-half stability would strengthen the case for person-level interpretation. Because the stability of the two slopes cannot be determined confidently a priori, we treat it as an open empirical question.

Recent methodological work reinforces this requirement. Random or Monte Carlo split-half procedures reduce dependence on a single arbitrary partition (Pronk et al., 2022), while correlations among cognitive-task scores can be strongly influenced by trial noise and task design (Rouder et al., 2023). Task design for individual-differences research must therefore consider effect reproducibility, score reliability, and the range of task sampling simultaneously (Zorowitz & Niv, 2023; Brysbaert, 2024). Our 100 difficulty-balanced split-halves are intended precisely to separate process effects within the current task from the degree to which person-level scores can be treated as stable across item samples.

### 1.5. Reading Purposes, Comprehension Processes, and Cross-System Transportability

PIRLS distinguishes two reading purposes—literary experience and acquiring and using information—and reports separate scales for retrieving/making straightforward inferences and interpreting/integrating/evaluating (Mullis & Martin, 2019; Mullis et al., 2023). Large-scale evidence also suggests that metacognitive reading strategies can carry predictive information across educational contexts (Ghimire & Mokhtari, 2025). If demand-sensitive allocation primarily supports a specific higher-order process, criterion associations should be concentrated in selected subscales. If it reflects a more general task-management process, associations should be similar across reading purposes and comprehension processes. The 25-system design also permits a distinction between directional transportability and effect-size homogeneity. A common direction may be widespread even when effect magnitudes vary because of language, curriculum, digital experience, or test-taking culture. Random-effects meta-analysis and leave-one-system-out analyses allow these two properties to be evaluated separately.

### 1.6. Research Questions and Theoretical Expectations

To distinguish directional theoretical expectations from open empirical questions, we formulate expectations only when theory implies a clear direction. Questions concerning effect form, between-system heterogeneity, person-level stability, and cross-item generalization are posed as research questions. Research questions are intended to receive empirical answers rather than to be accepted or rejected as if they were directional hypotheses.

Theoretical Expectation 1 (task responsiveness) is as follows: If first-visit time is behaviorally responsive to increasing task demand, higher cross-fitted empirical item difficulty should be associated with longer first-visit time, and this direction should replicate with relatively high consistency across education systems. This expectation establishes whether first-visit time tracks the empirical difficulty gradient; it does not by itself identify the cognitive process responsible for longer time or treat the observation that difficult items take longer as the theoretical contribution.

**RQ1.**  *Does item difficulty systematically alter the probability of revisiting? If so, how consistent are the direction, magnitude, and between-system heterogeneity of this association?*

Theoretical Expectation 2 (criterion association) is as follows: After controlling mean first-visit time, overall revisit rate, and student background, demand-sensitive first-visit time allocation observed within the current assessment context is expected to be positively associated with PIRLS reading achievement. This expectation concerns criterion association and does not assume high person-level stability across item samples.

Theoretical Expectation 3 (criterion association of re-engagement) is as follows: After controlling mean first-visit time, overall revisit rate, and student background, demand-sensitive revisiting is expected to be positively associated with PIRLS reading achievement. Because revisiting involves additional monitoring, disengagement, and re-entry decisions, its effect size is not assumed to match that of time adaptation.

**RQ2.**  *Are the associations of time adaptation and revisit adaptation with overall reading, literary reading, informational reading, retrieving/making straightforward inferences, and interpreting/integrating/evaluating broadly similar across reading purposes and comprehension processes, or do they show clear process specificity?*

**RQ3.**  *How transportable are the directions of the two process–achievement associations across 25 education systems, and to what extent can directional consistency coexist with heterogeneity in effect magnitude?*

**RQ4.**  *As person-level process indicators, how stable are time adaptation and revisit adaptation across difficulty-balanced but nonoverlapping item subsets, and is that stability sufficient to support trait-like individual-differences interpretation?*

**RQ5.**  *When process indicators and outcomes are constructed from mutually nonoverlapping item sets, do the two demand-sensitive process indicators remain associated with held-out item performance, and do time adaptation and revisit adaptation show comparable strength of cross-item generalization?*

## 2. Materials and Methods

### 2.1. Data Source and Sample

Data came from the PIRLS 2021 International Database, Edition 2, including the student background and achievement file (ASG), the item-response file (ASA), and the process-indicator file (ASP) (IEA, 2024; Fishbein et al., 2024). The initial audit covered 26 candidate digitalPIRLS education systems. The public ASP files share a common variable structure: 273 items provide total time (*_S), first-visit time (*_R), and number of visits (*_F), and an additional 91 items provide first-visit time only. All 273 items with complete process information could be matched item-by-item to corresponding variables in ASA. The U.S. R5 ASP file retained the same fields but contained no valid nonmissing values on the core process variables and was therefore excluded from process analyses. The final analytic scope comprised 25 education systems with usable process data.

Across these 25 systems, the ASP files contained 158,142 student records. Of these, 36,234 had no nonmissing core process record (*_S, *_R, or *_F) and could not enter the process-and-score matching stage. All remaining 121,908 students had at least one scored item with process information and a finite cross-fitted difficulty value; this is the process-record sample in Table 1. No additional student was lost solely because a scored item could not be matched or because all cross-fitted difficulties were unavailable. Students contributed an average of 27.63 matched scored-item records with finite difficulty. This count does not require valid values for every process field or a valid person-specific slope. The study used only publicly available, de-identified secondary data.

Within the process-record sample, 121,078 students had a valid time-adaptation indicator and 121,615 had a valid revisit-adaptation indicator. The primary M2 specification required both indicators: 538 students lacked only time adaptation, one lacked only revisit adaptation, and 292 lacked both, leaving 121,077 with the required process predictors. Missing primary background covariates then excluded 4871 students, leaving exactly 116,206 M2 complete cases (95.3% of the process-record sample). Table 1 reports each of these student-level reasons separately for every system. These categories follow the actual processing sequence and are additive: initial ASP N minus no-process records yields process-record N, and process-record N minus the three slope-unavailability categories and subsequent background exclusions yields M2 N.

The process-field plausibility rules (1–1800 s for time and 1–30 for visits) set individual out-of-range values to missing; they do not automatically exclude an entire student. A cross-fitted difficulty required at least 40 training responses, and a person-specific slope required at least eight valid items and four distinct difficulty values. In all 25 systems, the difficulty requirement caused no further student-level exclusion after matching. There were no additional M2 exclusions due to missing plausible values or design variables after the process and background requirements were met. M2 included sex, reading self-confidence, digital self-efficacy, and liking reading, alongside the process predictors; home resources and parental education were not separate M2 covariates. Home resources (ASBGHRL) entered only the M3 sensitivity model.

### 2.2. Process Indicators and Item Scoring

In ASP, first-visit time denotes the duration of a student’s first entry into an item, total time denotes cumulative time across all visits to the item, and number of visits indicates how many times the item was entered. First-visit and total times were retained when they fell between 1 and 1800 s, and visit counts were retained between 1 and 30. These deliberately broad bounds were used as data-quality plausibility filters to remove zero/negative values and extreme or likely recording anomalies; they were not treated as substantive thresholds for efficient or inefficient reading. Time variables were transformed using log(1 + x) to reduce right-skew. An item was coded as revisited when the visit count exceeded 1; missing visit counts remained missing.

For multiple-choice items in ASA, raw values 1–4 correspond to options A–D. Correct options were parsed from item labels and responses were scored 0/1. Constructed-response items were divided by their maximum score to obtain proportional scores ranging from 0 to 1. Identifiable omitted or invalid responses were treated as incorrect (0), whereas not-reached or not-administered responses remained missing. Proportional scoring was used only to place item easiness on a common 0–1 directional scale when estimating the empirical difficulty gradient and held-out item performance; the principal achievement outcomes remained the official PIRLS plausible values. Thus, differences in raw scoring range between multiple-choice and constructed-response items did not create different achievement scales in the criterion models.

### 2.3. Five-Fold Cross-Fitted Empirical Demand Gradient

Within each education system, students were randomly partitioned into five folds using a fixed reproducibility seed (20260826, offset by education system). For students in fold k, item difficulty was estimated only from students in the other four folds. Training samples used the PIRLS total weight (TOTWGT) to compute weighted mean proportional item scores; difficulty was defined as 1 minus weighted easiness, with at least 40 training responses required per item. Difficulty values were then standardized using the mean and standard deviation of the training-set item difficulties. A target student’s own item responses therefore did not contribute to the difficulty values used to construct that student’s process indicators, reducing direct information feedback from individual achievement into the predictor. We refer to this operational variable as a cross-fitted empirical demand gradient because it is derived from the same assessment and education system rather than from an external task-calibration study.

### 2.4. Person-Level Demand-Sensitive Process Indicators

Time adaptation was defined as each student’s within-person slope linking the standardized cross-fitted empirical difficulty gradient to log first-visit time. Larger slopes indicate that first-visit time increases more strongly as empirically estimated difficulty rises; they do not identify why the additional time was spent. Revisit adaptation was defined as the within-person linear-probability slope linking the same difficulty gradient to a binary revisit indicator. Person-specific slopes required at least eight valid items and at least four distinct difficulty values. At the group level, revisiting was additionally examined with a binomial logistic mixed model; the person-level linear-probability slope was used because it provides a directly comparable finite-item individual parameter.

Finite item sets introduce sampling noise into person-specific slopes. We therefore applied empirical-Bayes shrinkage based on the estimated slope and its standard error, pulling less precise estimates modestly toward the education-system mean. The shrunken indicators (time_adapt_eb and revisit_adapt_eb) were standardized within each education system and used as the primary person-level process measures. Empirical-Bayes shrinkage improves estimation precision but does not establish cross-item reliability; repeated split-half analyses were therefore used to evaluate person-level ranking stability directly.

### 2.5. Reading Outcomes and Covariates

Reading outcomes were represented by all five plausible values for overall reading (ASRREA01–05), literary reading (ASRLIT01–05), informational reading (ASRINF01–05), retrieving/making straightforward inferences (ASRRSI01–05), and interpreting/integrating/evaluating (ASRIIE01–05). Covariates included sex (ITSEX), reading self-confidence (ASBGSCR), digital self-efficacy (ASBGSEC), and liking reading (ASBGSLR). Continuous scales were standardized within education systems. An additional complete-case sensitivity model included home resources for learning (ASBGHRL); its results are reported directly in Section 3.3.

To reproduce the archived specification, sex was entered using the labeled factor produced by haven::as_factor (levels = “labels”). A labeled nonresponse category was retained where present; it contributed one record in New Zealand. Continuous background scales used their defined missing-value codes. The M2 N therefore counts the records actually used by the fitted specification, including that retained factor category.

### 2.6. Main Analyses

Item-level behavioral associations were estimated separately within each education system. First-visit time was modeled with a cross-classified linear mixed model including standardized item difficulty and item maximum score as fixed effects, with random intercepts for students and items. Revisiting was modeled with a binomial logistic mixed model using the same fixed-effect structure. The difficulty coefficients from the 25 systems were synthesized using random-effects meta-analysis estimated by restricted maximum likelihood (REML).

Student-level criterion associations were estimated separately within each education system in R using the official PIRLS total weight (TOTWGT), Jackknife replicate-weight design information, and all five plausible values (von Davier et al., 2023). Replicate weights were generated from JKZONE and JKREP with ILSAstats::repcreateILSA (study = “PIRLS”, year = 2021, repwtname = “RWT”). Each weighted regression was then fitted with ILSAstats::replm(), specifying the five reading plausible values jointly (relatedpvs = TRUE), TOTWGT as the final student weight, the generated replicate weights, and method = “PIRLS”. For each coefficient, the five plausible-value estimates were averaged. Total variance combined the mean replicate-weight sampling variance (U-bar) with between-plausible-value variance (B) as T = U-bar + (1 + 1/5)B = U-bar + 1.2B. The resulting system-specific coefficient and standard error were then entered into the cross-system REML random-effects meta-analysis implemented with metafor::rma.uni(). Baseline models included mean first-visit time, overall revisit rate, and student background variables; the primary models additionally included within-system standardized time adaptation and revisit adaptation. We report pooled estimates, 95% confidence intervals and I-squared and use leave-one-education-system-out analyses to evaluate whether the pooled direction depends on any single system. The system-specific overall-reading estimates and the pooled estimates were derived from the same primary M2 analyses.

For sample-size verification, the original scoring, value-cleaning, cross-fitting, slope, shrinkage, and standardization steps were reconstructed from the public R5 files. Within each system, all five overall-reading plausible values used the same complete-case student set. The five weighted-regression coefficients were averaged, reproducing both archived M2 process coefficients in all 25 systems to within 10^−10^. The verified M2 sample sizes correspond to the students included in the primary regressions underlying the archived coefficients and their plausible-value/Jackknife confidence intervals. These are model sample sizes rather than the larger process-record counts.

### 2.7. Model Diagnostics, Cross-Item Stability, Booklet Sensitivity, and Item-Set Holdout

For model diagnostics, saved objects from the 25 time models and 25 revisit models were inspected for optimizer return status, convergence messages, and lme4 isSingular flags. Random-effect variances were also recorded to ensure that student- and item-level components had not collapsed to zero. The saved objects did not contain usable derived gradient or Hessian quantities; accordingly, we do not claim gradient- or Hessian-based diagnostics.

Cross-item stability was evaluated using 100 repeated split-halves. Within each booklet, items were first stratified by difficulty and then randomly assigned to halves A and B while balancing item count and difficulty structure. Each half required at least six valid items. Raw and empirical-Bayes slopes were re-estimated separately in the two halves, half-score correlations were computed, and Spearman–Brown correction was applied for test length. For each education system, we report the median and 2.5th–97.5th percentile interval across 100 repetitions. The empirical-Bayes Spearman–Brown coefficient is treated as the primary stability index. Repeated random partitioning was preferred to a single odd–even or first–second-half split because the partitioning rule itself can materially alter split-half estimates in cognitive tasks (Pronk et al., 2022; Parsons et al., 2019).

Booklet sensitivity was examined in two ways. First, booklet fixed effects were added to the official PIRLS student-level models while retaining the original within-system standardized process indicators. Second, time adaptation, revisit adaptation, mean first-visit time, and overall revisit rate were re-standardized within booklet before fitting models that also included booklet fixed effects. Both specifications used TOTWGT, Jackknife replicate weights, and all plausible values. These analyses tested whether the primary associations were explained by systematic differences in booklet composition.

Finally, we conducted five-fold item-set holdout analyses to reduce coupling created when process indicators and achievement share the same items. Within each booklet, items were divided into five mutually exclusive sets. In each iteration, four fifths of the items were used to estimate time adaptation, revisit adaptation, mean first-visit time, and overall revisit rate; these source-set indicators were then used to explain performance on the remaining fifth. Held-out items never contributed to the corresponding student’s process slopes. The primary model used proportional item score (0–1) as the outcome, controlled source-set mean first-visit time, source-set overall revisit rate, item maximum score, holdout fold, and booklet and included random intercepts for students and items. All 25 education systems completed this all-scored-item model. As an additional sensitivity analysis, an MC-only binomial logistic model was completed for 15 systems; this subset was not used as the basis for the primary cross-system inference.

## 3. Results

### 3.1. Difficulty and First-Visit Time: A Highly Consistent Task Response

The results were consistent with Theoretical Expectation 1. The difficulty–first-visit-time coefficient was positive in all 25 education systems. The random-effects pooled coefficient was b = 0.1057, SE = 0.0065, 95% CI [0.0929, 0.1184], *p* < .001, I^2^ = 51.97% (Table 2). Because the outcome was log-transformed, this coefficient corresponds approximately to an 11.1% increase in first-visit time for each 1-SD increase in cross-fitted empirical item difficulty. The result establishes that first-visit time reliably covaries with the empirical difficulty gradient; it does not by itself identify the specific cognitive process that produced the longer time.

### 3.2. Difficulty and Revisiting: A Weak Average Effect

For RQ1, item difficulty showed only a weak and clearly system-dependent association with revisiting. The pooled log-odds coefficient was b = 0.0196, SE = 0.0100, 95% CI [−0.0001, 0.0392], *p* = .0506, I^2^ = 64.50%, corresponding to an odds ratio of approximately 1.020. Only 13 of 25 system-specific point estimates were positive. Thus, increasing item difficulty did not produce a revisit response as consistent as the first-visit-time response; re-engagement appears more contingent on specific task conditions, monitoring decisions, and system context.

### 3.3. Criterion Associations with Reading Achievement

The results were consistent with Theoretical Expectations 2 and 3, although the magnitudes differed sharply between the two process indicators. After controlling mean first-visit time, overall revisit rate, sex, reading self-confidence, digital self-efficacy, and liking reading, a 1-SD increase in time adaptation was associated with a 19.92-point advantage in overall reading, 95% CI [17.84, 22.00], *p* < .001 (Table 3). Point estimates were positive in all 25 systems and ranged from approximately 13.70 to 37.62 points (Table 4). A 1-SD increase in revisit adaptation was associated with a 3.19-point advantage in overall reading, 95% CI [2.29, 4.09], *p* < .001. The pooled time-adaptation coefficient should be read as a cross-system average rather than a universal effect size: between-system heterogeneity was very high (I^2^ = 93.97%), and the system-specific magnitudes varied substantially.

The two indicators therefore showed a pronounced asymmetry in criterion association. Time adaptation was substantially more strongly related to achievement than revisit adaptation. Because the models controlled average first-visit time and the overall revisit rate, these coefficients reflect incremental information carried by the coupling between empirical item difficulty and process behavior, rather than a simple association of slow reading or frequent revisiting with higher achievement. In an additional complete-case sensitivity analysis (88,504 overall-reading complete cases across 25 systems) that also controlled home resources for learning (ASBGHRL), the pooled overall-reading association remained positive for both indicators but was attenuated: time adaptation b = 18.05, 95% CI [16.12, 19.99], and revisit adaptation b = 2.76, 95% CI [2.03, 3.49]. The same pattern held for literary reading (time b = 18.43, 95% CI [16.37, 20.48]; revisit b = 3.02, 95% CI [2.33, 3.70]), informational reading (time b = 18.12, 95% CI [16.23, 20.01]; revisit b = 2.73, 95% CI [1.98, 3.49]), retrieving/making straightforward inferences (time b = 18.39, 95% CI [16.51, 20.27]; revisit b = 2.77, 95% CI [2.10, 3.44]), and interpreting/integrating/evaluating (time b = 18.17, 95% CI [16.11, 20.23]; revisit b = 2.67, 95% CI [1.96, 3.37]). Thus, adding home resources modestly attenuated the estimates but did not reverse their direction or alter the marked asymmetry between the two process indicators. 

### 3.4. Consistency Across Reading Purposes and Comprehension Processes

For RQ2, the pattern was more consistent with broad cross-domain similarity than with a higher-order-process-specific effect. The pooled time-adaptation effects for literary reading, informational reading, retrieving/making straightforward inferences, and interpreting/integrating/evaluating were all close to 20 PIRLS points; revisit-adaptation effects were approximately 3 points across subscales. The similarity of estimates across outcome domains suggests that the information carried by these indicators is more plausibly situated at a task-resource-regulation level that operates across specific PIRLS reading subscales.

### 3.5. Directional Transportability and Heterogeneity in Effect Magnitude

RQ3 revealed a cross-system structure in which directional stability and effect-size heterogeneity coexisted. The time-adaptation association with overall reading showed very high between-system heterogeneity (I^2^ = 93.97%), and I^2^ exceeded 92% for the other reading outcomes. Accordingly, the pooled coefficients summarize an average relation across systems and should not be assumed to describe the magnitude in any particular education system. Directional stability was nevertheless strong: all 25 system-specific coefficients for overall reading were positive, and all 25 leave-one-system-out pooled estimates remained positive, ranging from 19.09 to 20.19 points. Even after removing the system with the largest effect, the pooled association remained close to the original estimate. Table 4 presents all 25 system-specific overall-reading estimates and 95% confidence intervals, so the magnitude of cross-system variation is visible rather than compressed into I^2^ alone.

The M2 N in Table 4 is the actual overall-reading complete-case count for the jointly estimated time- and revisit-adaptation predictors. The two coefficients use the same students within each system, and the count is identical across the five plausible values. The larger process-record N and all subsequent exclusions are reported in Table 1.

### 3.6. Diagnostics of the Main Mixed Models

All 50 primary mixed models were successfully saved and passed the prespecified core diagnostics (Table 5). Optimizer status was normal for all 25 first-visit-time models and all 25 revisit models; no convergence messages were recorded, and no model was flagged as singular by isSingular. Student and item random-intercept variances were all greater than zero. For first-visit-time models, student random-effect variances ranged from 0.087 to 0.261 and item random-effect variances from 0.103 to 0.336. For revisit models, the corresponding ranges were 3.896–7.981 and 0.071–0.405. Because usable gradient and Hessian-derived quantities were not available in the saved objects, no stronger claim is made regarding those diagnostics.

### 3.7. Person-Level Cross-Item Stability: Strong Criterion Association Did Not Translate into High Trait-like Reliability

**Figure 1:**
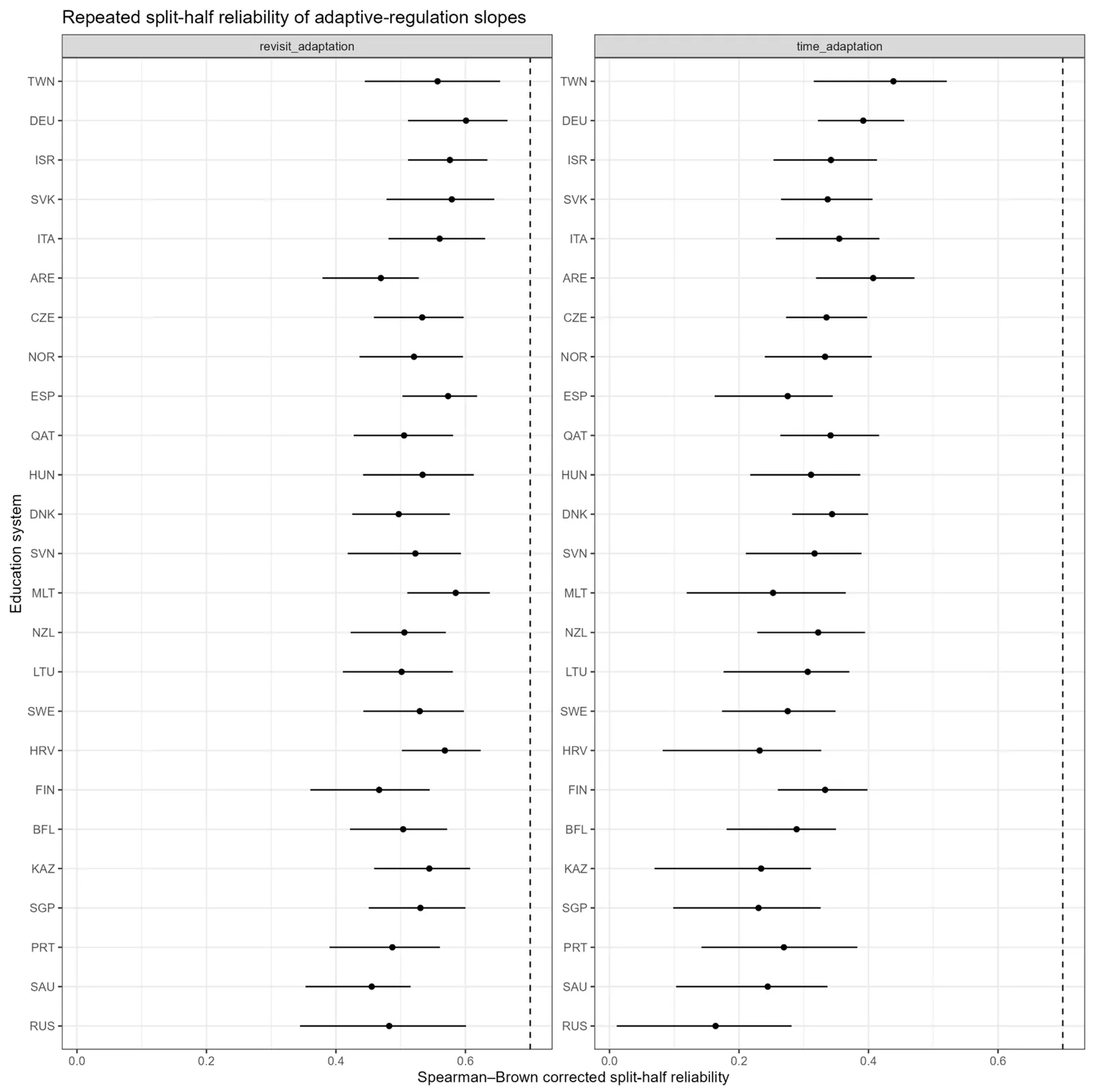
Repeated split-half reliability of demand-sensitive process slopes across 25 education systems.

RQ4 showed that neither indicator had cross-item stability sufficient to support a high-reliability trait interpretation, with the limitation especially pronounced for time adaptation. Across 100 difficulty-balanced split-halves, the cross-system median empirical-Bayes Spearman–Brown reliability for time adaptation was 0.317, with system-specific medians ranging from 0.164 to 0.438. Revisit adaptation was higher, with a cross-system median of 0.529 and system-specific medians ranging from 0.455 to 0.601. No system reached the 0.70 reference line shown in Figure 1. This finding does not negate the process effect; it directly constrains the universe over which person-level scores can be generalized.

The reliability results materially change the strength of individual-differences interpretation. Time adaptation was strongly associated with reading achievement, yet relative person rankings were only weakly preserved when students were scored on a different difficulty-balanced item subset (Table 6). Revisit adaptation was more stable but remained in a moderate range. Both indicators are therefore better interpreted as task-responsive process measures that contain person-level variation than as highly reliable, broadly stable trait scores.

Points show the median empirical-Bayes Spearman–Brown corrected reliability for each system across 100 repetitions; horizontal lines indicate the 2.5th–97.5th percentile interval. The dashed 0.70 line is shown only as a common reference for person-level score interpretation.

### 3.8. Booklet Sensitivity: Main Criterion Associations Were Robust to Booklet Adjustment

Adding booklet fixed effects did not attenuate the primary associations. For overall reading, the pooled time-adaptation effect was 21.61 points, 95% CI [19.21, 24.00], and the revisit-adaptation effect was 3.30 points, 95% CI [2.40, 4.20], both *p* < .001. After re-standardizing process indicators within booklet, time adaptation remained associated with 20.72 points, 95% CI [18.42, 23.01], and revisit adaptation with 3.84 points, 95% CI [2.95, 4.73]. Literary reading, informational reading, retrieving/making straightforward inferences, and interpreting/integrating/evaluating showed the same pattern.

The booklet-adjusted estimates remained close to the primary estimates (Table 7), so these sensitivity analyses did not show material attenuation of the main criterion associations after booklet composition was controlled. Importantly, booklet sensitivity and cross-item reliability address different problems. The former evaluates whether the average associations persist after booklet adjustment, whereas the latter shows that person-level process scores remain sensitive to the particular items sampled. Together, these analyses delimit the interpretation of the indicators more precisely.

### 3.9. Item-Set Holdout: Limited Person-Level Stability Coexisted with Cross-Item Criterion Information

RQ5 received an affirmative answer, but the two indicators were clearly asymmetric. In the five-fold item-set holdout, four fifths of the items within each booklet were used to estimate process indicators, and the remaining nonoverlapping fifth was used to evaluate criterion association. For time adaptation, coefficients from the all-scored-item linear mixed model were positive in all 25 education systems and exceeded |t| > 1.96 in all 25. The random-effects pooled estimate was b = 0.0300, SE = 0.00125, 95% CI [0.0276, 0.0325], *p* < .001, I^2^ = 87.18%. Because held-out performance was scored on a 0–1 scale, a 1-SD increase in source-set time adaptation was associated with an average increase of approximately 3.0 percentage points in held-out item performance.

Revisit adaptation also retained a positive cross-item association, but with substantially weaker cross-system consistency: b = 0.00539, SE = 0.00062, 95% CI [0.00417, 0.00661], *p* < .001, I^2^ = 47.26%. Point estimates were positive in 23 of 25 systems and exceeded |t| > 1.96 in 13. All 25 all-scored-item holdout models optimized successfully, with no singular fits or convergence messages. Thus, the answer to RQ5 is not that both indicators generalize equally strongly. Rather, time adaptation shows highly consistent cross-item association, whereas revisit adaptation retains weaker and less system-consistent cross-item information. The limited person-level rank stability revealed by split-half analysis therefore should not be interpreted as an absence of cross-item criterion information.

As an additional sensitivity analysis, MC-only logistic models were completed in 15 systems. Time adaptation was positive and significant in all 15, with a pooled log-odds coefficient of 0.3035, corresponding to OR = 1.355, 95% CI [1.319, 1.391]. Revisit adaptation yielded a pooled OR = 1.048, 95% CI [1.037, 1.059] (Table 8). Because the MC-only analysis was completed in only 15 systems and this subset was not randomly selected for independent validation, it is treated as additional robustness evidence rather than as the basis for the primary 25-system holdout inference.

## 4. Discussion

### 4.1. Core Finding: Strong Criterion Association, Limited Person-Level Stability, and Cross-Item Generalization Coexisted

The five research questions did not produce a uniform pattern of positive confirmation. RQ1 showed that the average revisit response to difficulty was weak and system-dependent; RQ2 indicated broadly similar criterion associations across reading purposes and comprehension processes; RQ3 showed highly stable directions but markedly heterogeneous effect magnitudes; RQ4 revealed only limited-to-moderate person-level cross-item stability; and RQ5 showed strong within-assessment cross-item generalization for time adaptation but weaker generalization for revisit adaptation. This asymmetry shifts the focus away from treating the indicators as direct measures of a single stable ability and toward specifying which inferences are supported by observable task-conditioned behavior.

The central contribution comes from the joint pattern across three forms of evidence. First, demand-sensitive first-visit time allocation showed a very strong cross-system criterion association with reading achievement: all 25 systems had positive effects, and pooled effects across the five PIRLS reading outcomes were close to 20 points. Second, 100 repeated split-halves showed that person rankings on time adaptation were only weakly stable across item subsets, with a median Spearman–Brown reliability of approximately 0.32. Third, when the process slope was estimated from four fifths of the items and achievement was evaluated on a completely nonoverlapping fifth, time adaptation remained positive and significant in all 25 systems, with an average difference of about three percentage points in held-out proportional score per SD of the source-set indicator. Taken together, these results show that time adaptation is neither well characterized as a highly reliable trait score nor dismissible as noise simply because its cross-item ranking reliability is limited.

Strong criterion association, limited reliability, and cross-item generalization are not statistically contradictory. Split-half reliability asks whether relative person ranking is preserved when the sampled items change. Holdout analysis asks whether a task-response pattern estimated from one item set remains associated with performance on another item set. The former sets a high bar for person-level diagnostic use; the latter addresses whether the process indicator carries information that generalizes beyond its source items. The present findings show that time adaptation is sensitive to item sampling at the level of person ranking while still retaining stable cross-item criterion information. Process-data research should therefore distinguish at least three claims: an indicator is informative within the current task, person rankings are stable across items, and criterion association generalizes across nonoverlapping items.

This configuration extends the reliability-paradox literature. Prior work has emphasized that robust group-level effects do not guarantee reliable individual-differences scores (Hedge et al., 2018; Enkavi et al., 2019). The present item-set holdout adds an important qualification: low cross-item rank stability does not imply that a process indicator is devoid of criterion information beyond its source items. Stable ranking of individuals and transfer of predictive information from one item set to another are distinct measurement questions.

### 4.2. Time Adaptation and Revisit Adaptation Showed an Asymmetry Between Criterion Association and Stability

The two indicators also displayed a theoretically informative asymmetry. Time adaptation was far more strongly associated with reading achievement than revisit adaptation, yet its split-half stability was lower. Revisit adaptation was associated with roughly 3 PIRLS points and had a median split-half reliability of about 0.53. This pattern indicates that criterion magnitude and trait-like stability are distinct dimensions. Initial time allocation may respond closely to local task demand and therefore track current cognitive load more strongly, while also being more sensitive to specific item content. Revisiting requires monitoring, disengagement, and re-entry and may contain a stronger general behavioral-style component, producing greater cross-item ranking stability but less incremental association with reading performance.

The asymmetry is also compatible with theories of cognitive effort allocation. Cognitive control is regulated by both demand and cost–benefit considerations (Shenhav et al., 2017; Kool & Botvinick, 2018). Initial time can respond rapidly to local demand, which may yield stronger immediate criterion association while leaving it more vulnerable to item-specific variation. Revisiting involves an additional decision to return, which may reflect more stable monitoring or action tendencies. This interpretation is consistent with the present pattern—stronger criterion association for time and greater stability for revisiting—but should be tested with independent measures of cognitive control and metacognition.

The item-level results reinforce this distinction. Difficulty had a positive effect on first-visit time in every education system, indicating that increased initial processing is a widespread response to higher task demand. The average difficulty effect on revisiting, by contrast, was near the conventional significance boundary and showed mixed directions across systems. Student-level revisit adaptation nevertheless remained positively associated with achievement, suggesting that its informative component lies in whether revisiting is selectively directed toward items that require additional processing rather than in the amount of revisiting itself.

### 4.3. Implications for Measuring Cognitive Processes and Individual Differences

These findings speak directly to the broader use of process data in research on cognition and individual differences. Digital assessments increasingly make time, clicks, returns, and navigation traces technically easy to extract, but technical availability does not make a measure suitable for individual diagnosis or uniquely identify the mental process that generated it. The present results show that low split-half reliability and significant cross-item criterion association can coexist. The former constrains the use of a single-test slope for stable person ranking; the latter supports using the indicator to study task-conditioned behavioral regularities and their relation to achievement. When the research aim is person diagnosis or cross-situational trait inference, more items, repeated assessments, independent tasks, and convergent process measures are required. This distinction is consistent with recent cognitive-ability research emphasizing that within-person performance variability can carry theoretical information that is obscured by a single summary score (Vaughan & Birney, 2023).

From a measurement perspective, the results reinforce an emerging methodological consensus: reliability and criterion-related evidence for cognitive tasks must be reported and interpreted separately. Recent reviews and tutorials show that behavioral tasks often display limited reliability, and recommended remedies include increasing informative trials, optimizing the stimulus range, using hierarchical models, and redesigning tasks to enlarge meaningful between-person variability (Zorowitz & Niv, 2023; Brysbaert, 2024). The present results further suggest that process slopes require explicit specification of their universe of generalization. If the goal is to characterize a group-level behavioral relation or cross-item criterion association, the current indicators are informative; if the goal is person-level diagnosis, the current item count is insufficient.

Demand-sensitive slopes are therefore better treated as process markers than as replacements for conventional ability scales. They can describe how observed behavior changes as task demand changes, contribute to theory-driven process models, and provide incremental criterion information. Developing them into individual-differences measurement tools would require more items, broader task domains, repeated measurement, or multilevel random-slope models that decompose variance attributable to person, item, text, booklet, and occasion.

### 4.4. Broad Criterion Associations and Cross-Item Generalization Do Not Establish a General Intelligence Trait

Time adaptation showed similar associations with literary reading, informational reading, retrieving/making straightforward inferences, and interpreting/integrating/evaluating, and item-set holdout showed that its criterion association did not depend on including the outcome items in the slope construction. Together, these findings support the presence of broadly relevant task-related information. They do not, however, establish that time adaptation is a measure of general intelligence or a stable executive-function trait. The split-half results show that its person-level realization remains strongly constrained by the sampled item set.

A more defensible theoretical location is between task demand and specific comprehension performance: demand-sensitive time allocation captures how observed first-visit time changes as current task conditions change and may reflect demand-sensitive differences in resource investment. It may be influenced jointly by general cognitive ability, reading knowledge, metacognitive monitoring, executive control, and experience with digital tasks. Independent measures of working memory, fluid intelligence, cognitive reflection, or executive function will be needed before stronger claims can be made about its relation to broader intelligence structure.

### 4.5. Directional Stability and Magnitude Heterogeneity Can Coexist Across Education Systems

Across 25 education systems, the direction of the time-adaptation association with overall reading was highly consistent, and leave-one-system-out analyses never reversed the positive pooled association. At the same time, the very high I^2^ values show that effect magnitude varied markedly. The pooled estimate should therefore not be treated as a common effect that applies equally to all education systems. Directional consistency is compatible with a broadly recurring behavioral relation, whereas magnitude differences may reflect language, curriculum, item characteristics, digital experience, test familiarity, or sample composition. These explanations remain hypotheses for future moderator analyses rather than conclusions from the present data.

Booklet sensitivity did not support simple booklet composition as an explanation for the main associations. Adding booklet fixed effects and re-standardizing indicators within booklet left the time- and revisit-adaptation associations largely unchanged. These results concern the robustness of the average associations; they do not explain the substantial between-system heterogeneity. Prespecified system-level moderator analyses are needed in future research.

### 4.6. Contributions to RESOLV and Research on Reading Regulation

The present study provides a scalable process operationalization for task-oriented reading theory. RESOLV proposes that task representation guides reading action; by using cross-fitted empirical item difficulty as the demand gradient, the current design tests whether observable reading behavior changes systematically with estimated task difficulty. First-visit time responded consistently to this gradient, and person-specific slopes were strongly associated with reading performance. These patterns are compatible with demand-sensitive allocation accounts, but first-visit time cannot distinguish attentional investment from decoding difficulty, strategy use, interface effects, or other processes. Reliability evidence further requires the interpretation to remain task-conditional: the slope currently resembles a task-responsive behavioral process indicator more than an ability that remains stable independently of specific items.

Integrating RESOLV with broader cognitive-effort theory further clarifies the construct. RESOLV addresses why task representations alter reading actions, whereas effort-allocation theories address why resource investment must be adjusted dynamically as a function of expected benefit and cost (Shenhav et al., 2017; Kool & Botvinick, 2018). Together, these accounts suggest that selectively adjusting time investment to current demand may support performance, although the present observational findings do not establish a causal benefit.

A second contribution is the separation of initial allocation from re-engagement. First-visit time and revisiting showed different group-level behavioral patterns, criterion associations, and cross-item stability, demonstrating that a broad engagement label can conceal meaningful process distinctions. A third contribution concerns validation logic itself: cross-fitted difficulty reduces direct feedback of a student’s own performance into the demand indicator; booklet sensitivity did not support a simple composition account; split-half analysis limits trait inference; and item-set holdout further separates process construction from outcome items. Together, these forms of evidence support the narrower but more defensible interpretation of a task-responsive behavioral process indicator.

### 4.7. Educational Implications

The practical implication is better framed as demand recognition than as simply increasing reading time. Requiring all students to slow down would increase time costs across items without ensuring that resources are concentrated where they are needed. More targeted instruction would help students detect changes in task demand, judge whether evidence is sufficient, and increase initial processing or checking when warranted. Digital platforms could potentially combine item-demand information with ongoing behavior to identify cases in which difficult items receive unusually brief first visits and prompt students to re-examine task requirements.

The reliability findings also caution against assigning stable labels to individual students on the basis of a single time-adaptation score. At present, the indicator is better suited to group research, classroom process diagnosis, or trend analysis across repeated observations. Person-level feedback would require greater dependability through more items and repeated tasks.

### 4.8. Limitations and Future Research

First, this is an observational analysis of a large-scale assessment. Associations between process indicators and reading achievement should not be interpreted causally, and first-visit time should not be treated as a direct readout of cognitive resource allocation. Higher reading ability, language knowledge, executive function, metacognitive monitoring, strategy choice, decoding difficulty, or interface interaction may influence both observed time allocation and achievement. The background-adjusted, home-resource, and booklet sensitivity models did not show that these controls eliminated the observed associations, but they cannot identify which latent process generated the timing pattern or eliminate all common causes.

Second, the split-half results show clear item-sampling dependence in person-specific slopes. Empirical-Bayes shrinkage did not eliminate this limitation. Future studies should increase the number of valid items per student, include multiple texts, tasks, and occasions, and use generalizability-theory or multilevel random-coefficient approaches to decompose variance attributable to person, item, text, and occasion. This limitation is consistent with the broader individual-differences literature on behavioral tasks and underscores the need to expand the item universe, add repeated measurements, and optimize task sensitivity if time adaptation is to be developed into a stable person-level measure (Zorowitz & Niv, 2023; Brysbaert, 2024).

Third, the public ASP files provide item-level aggregate process indicators rather than fine-grained event sequences. First-visit time and number of visits characterize when and how long students interact with an item, but they cannot identify which part of the text was attended to, whether an answer was changed, whether extra time reflected productive evidence integration or decoding difficulty, or what search path was used. Raw event logs, eye tracking, or independent process measures would be needed to distinguish these candidate mechanisms more directly.

Fourth, although item-set holdout removes direct overlap between the items used to estimate process slopes and the items used as outcomes, source and held-out items still come from the same PIRLS assessment and booklet structure and may share passages, interface features, or testing context. The result should therefore be interpreted specifically as within-assessment cross-item generalization, not as predictive validity across independent tests, texts, occasions, or settings. Future work should use independent texts, separate testing occasions, or external cognitive tasks to evaluate broader transportability.

Fifth, the MC-only holdout logistic models were computationally intensive and were completed for 15 systems only. Their direction was consistent with the 25-system all-scored-item results, but the 15 systems were not randomly selected for independent validation. We therefore do not use the MC-only results as the primary cross-system evidence; the core holdout conclusion rests on the all-scored-item model completed in all 25 systems.

Sixth, between-system heterogeneity was high. The present analyses were designed primarily to establish directional transportability rather than explain heterogeneity. Future work should preregister meta-regression models using theoretically specified system-level predictors such as digital-instruction experience, curriculum characteristics, assessment language, or socioeconomic composition, thereby reducing the risk of post hoc moderator selection.

## 5. Conclusions

digitalPIRLS 2021 reveals a clear task-responsive pattern in digital reading behavior. As the cross-fitted empirical difficulty gradient increased, students consistently spent more time on their first item visit; students whose first-visit time tracked difficulty more strongly also showed substantially higher reading performance across five PIRLS outcomes. Within-assessment item-set holdout analyses further showed that this association did not depend on using the same items to construct the process slope and the criterion: time adaptation estimated from four fifths of the items remained positively and significantly associated with performance on the remaining nonoverlapping fifth in all 25 education systems. Revisiting represented a second, weaker re-engagement pathway, with a positive cross-item association but lower cross-system consistency than time adaptation. Effect magnitudes, however, varied substantially across education systems, so the pooled coefficients should be interpreted as averages rather than universal system-level effects.

Measurement evidence simultaneously limits trait-level and mechanistic interpretation. Despite strong criterion associations and clear within-assessment cross-item generalization, the median repeated split-half reliability of time adaptation was only about 0.32 across systems, compared with about 0.53 for revisit adaptation. Booklet controls did not attenuate the criterion associations, but neither the reliability analyses nor the process traces identify a unique cognitive mechanism. Demand-sensitive process slopes are therefore best interpreted as task-responsive behavioral indicators that are consistent with resource-allocation accounts: they describe how observed time and revisiting change with empirically estimated item difficulty and carry criterion information across items, but the available evidence does not support their use as highly stable trait scores, stand-alone diagnostic measures, or direct measures of cognitive resource allocation. More broadly, task responsiveness, criterion association, person-level stability, cross-item generalization, and mechanistic specificity should be evaluated separately when assigning psychological meaning to digital process indicators.

## Figures and Tables

**Table 1:** System-level sample flow and student-level reasons for exclusion.

Education System	Initial ASP N	No Core Process Records	Process-Record N	Time Slope Missing Only	Revisit Slope Missing Only	Both Slopes Missing	Missing M2 Controls	M2 N
Belgium (Flemish)	5114	1170	3944	9	0	5	104	3826
Chinese Taipei	5555	1260	4295	23	0	2	26	4244
Croatia	3937	880	3057	6	0	2	70	2979
Czech Republic	6621	1544	5077	15	0	2	143	4917
Denmark	4821	1113	3708	3	0	16	130	3559
Finland	7018	1583	5435	12	0	5	78	5340
Germany	4611	1070	3541	25	0	7	602	2907
Hungary	5312	1191	4121	3	0	2	74	4042
Israel	4890	1144	3746	43	0	29	121	3553
Italy	5440	1259	4181	7	0	10	60	4104
Kazakhstan	7023	1660	5363	9	0	9	136	5209
Lithuania	4623	1029	3594	17	0	0	91	3486
Malta	3030	675	2355	5	0	4	65	2281
New Zealand	5557	1261	4296	12	0	19	218	4047
Norway	5382	1224	4158	10	0	9	156	3983
Portugal	6111	1374	4737	10	0	5	54	4668
Qatar	5258	1237	4021	56	0	29	184	3752
Russian Federation	5217	1162	4055	2	0	5	75	3973
Saudi Arabia	4778	1166	3612	17	0	17	698	2880
Singapore	6719	1502	5217	10	0	2	49	5156
Slovak Republic	4841	1117	3724	6	0	8	113	3597
Slovenia	5110	1172	3938	7	0	2	88	3841
Spain	8551	1950	6601	27	0	20	173	6381
Sweden	5175	1202	3973	22	0	12	145	3794
United Arab Emirates	27,448	6289	21,159	182	1	71	1218	19,687
Total (25 systems)	158,142	36,234	121,908	538	1	292	4871	116,206

**Table 2:** Random-effects meta-analysis of item difficulty and process behavior.

Process Outcome	k	b	SE	95% CI	*p*	I^2^	Direction
Log first-visit time	25	0.1057	0.0065	[0.0929, 0.1184]	<.001	51.97%	25/25 positive
Revisit probability (log-odds)	25	0.0196	0.0100	[−0.0001, 0.0392]	.0506	64.50%	13/25 positive

**Table 3:** Demand-sensitive process indicators and PIRLS reading achievement: 25-system random-effects meta-analysis.

PIRLS Outcome	Process Indicator	k	Estimate	95% CI	*p*	I^2^
Overall reading	Time adaptation	25	19.92	[17.84, 22.00]	<.001	93.97%
Overall reading	Revisit adaptation	25	3.19	[2.29, 4.09]	<.001	68.46%
Literary reading	Time adaptation	25	20.42	[18.20, 22.65]	<.001	93.70%
Literary reading	Revisit adaptation	25	3.45	[2.55, 4.35]	<.001	63.17%
Informational reading	Time adaptation	25	19.97	[17.91, 22.04]	<.001	93.47%
Informational reading	Revisit adaptation	25	3.12	[2.23, 4.00]	<.001	66.07%
Retrieving/straightforward inference	Time adaptation	25	20.27	[18.23, 22.30]	<.001	92.80%
Retrieving/straightforward inference	Revisit adaptation	25	3.17	[2.32, 4.02]	<.001	61.98%
Interpreting/integrating/evaluating	Time adaptation	25	20.10	[17.87, 22.34]	<.001	94.47%
Interpreting/integrating/evaluating	Revisit adaptation	25	3.06	[2.18, 3.94]	<.001	67.54%

**Table 4:** System-specific overall-reading associations from the primary M2 models.

Education System	M2 Complete-Case N	Time Adaptation b	95% CI	Revisit Adaptation b	95% CI
Belgium (Flemish)	3826	15.23	[12.81, 17.64]	5.51	[3.14, 7.88]
Chinese Taipei	4244	15.07	[12.65, 17.49]	3.01	[0.84, 5.17]
Croatia	2979	16.44	[13.55, 19.33]	2.00	[−0.80, 4.81]
Czech Republic	4917	15.94	[13.51, 18.38]	3.52	[1.18, 5.87]
Denmark	3559	18.36	[16.13, 20.60]	4.05	[1.63, 6.47]
Finland	5340	22.86	[20.22, 25.50]	4.39	[2.41, 6.37]
Germany	2907	20.39	[17.46, 23.31]	2.21	[−0.68, 5.10]
Hungary	4042	18.99	[16.57, 21.41]	6.37	[3.31, 9.43]
Israel	3553	22.57	[19.27, 25.86]	0.92	[−1.81, 3.64]
Italy	4104	17.31	[15.09, 19.53]	0.23	[−1.91, 2.37]
Kazakhstan	5209	21.91	[19.35, 24.48]	3.10	[0.74, 5.46]
Lithuania	3486	16.68	[14.33, 19.03]	0.84	[−2.02, 3.69]
Malta	2281	20.43	[17.00, 23.87]	2.22	[−1.40, 5.85]
New Zealand	4047	26.11	[23.37, 28.84]	5.00	[2.37, 7.62]
Norway	3983	20.21	[17.93, 22.49]	0.49	[−1.83, 2.81]
Portugal	4668	13.70	[11.77, 15.63]	0.57	[−1.65, 2.79]
Qatar	3752	27.56	[24.19, 30.92]	7.54	[3.84, 11.23]
Russian Federation	3973	14.12	[11.94, 16.31]	1.14	[−1.22, 3.50]
Saudi Arabia	2880	24.93	[20.75, 29.10]	7.53	[3.89, 11.17]
Singapore	5156	18.75	[16.15, 21.34]	4.08	[1.57, 6.58]
Slovak Republic	3597	18.58	[15.20, 21.95]	2.11	[−0.30, 4.53]
Slovenia	3841	16.87	[14.41, 19.34]	2.91	[0.82, 5.01]
Spain	6381	16.08	[13.60, 18.55]	3.23	[1.17, 5.29]
Sweden	3794	22.18	[19.33, 25.02]	0.26	[−2.67, 3.20]
United Arab Emirates	19,687	37.62	[35.73, 39.51]	8.43	[6.10, 10.76]

**Table 5:** Summary of diagnostics for the primary mixed models.

Model	n	Optimizer OK	Convergence Messages	Singular Fits	Student RE Variance	Item RE Variance
Log first-visit time	25	25/25	0	0	0.087–0.261	0.103–0.336
Revisit probability	25	25/25	0	0	3.896–7.981	0.071–0.405

**Table 6:** Person-level stability from repeated split-half analyses.

Indicator	Systems	Cross-System Median SB Across 100 Repetitions	Range of System Medians	Interpretation
Time adaptation	25	0.317	0.164–0.438	Limited cross-item stability
Revisit adaptation	25	0.529	0.455–0.601	Moderate cross-item stability

**Table 7:** Booklet sensitivity analyses: 25-system random-effects meta-analytic estimates (estimate [95% CI]).

PIRLS Outcome	Time + Booklet FE	Time: Within-Booklet Standardized	Revisit + Booklet FE	Revisit: Within-Booklet Standardized
Overall reading	21.61 [19.21, 24.00]	20.72 [18.42, 23.01]	3.30 [2.40, 4.20]	3.84 [2.95, 4.73]
Literary reading	22.19 [19.63, 24.75]	21.40 [18.95, 23.86]	3.60 [2.69, 4.50]	4.14 [3.22, 5.06]
Informational reading	21.46 [19.08, 23.83]	20.57 [18.28, 22.86]	3.19 [2.30, 4.08]	3.77 [2.88, 4.66]
Retrieving/straightforward inference	21.94 [19.59, 24.29]	21.01 [18.76, 23.26]	3.29 [2.43, 4.15]	3.79 [2.93, 4.66]
Interpreting/integrating/evaluating	21.93 [19.33, 24.53]	21.04 [18.54, 23.54]	3.20 [2.31, 4.08]	3.74 [2.83, 4.64]

**Table 8:** Random-effects meta-analysis of item-set holdout models.

Model/Outcome	Indicator	k	Estimate	95% CI	*p*	I^2^	Direction
All-scored-item LMM	Time adaptation	25	0.0300	[0.0276, 0.0325]	<.001	87.18%	25/25 positive; 25/25 |t| > 1.96
All-scored-item LMM	Revisit adaptation	25	0.00539	[0.00417, 0.00661]	<.001	47.26%	23/25 positive; 13/25 |t| > 1.96
MC-only GLMM (sensitivity)	Time adaptation	15	log-odds = 0.3035	OR = 1.355 [1.319, 1.391]	<.001	84.99%	15/15 positive and significant
MC-only GLMM (sensitivity)	Revisit adaptation	15	log-odds = 0.0467	OR = 1.048 [1.037, 1.059]	<.001	21.78%	15/15 positive; 9/15 significant

## Data Availability

Publicly available data were analyzed in this study. The PIRLS 2021 International Database, Edition 2, including the digitalPIRLS process/log-file data, is available from the IEA Data Repository (DOI: 10.58150/PIRLS_2021_edition_2_including_Log-file_data). The present study used the publicly released ASG, ASA, and ASP files. No new primary data were collected for this study.
